# Characterization of MCF mammary epithelial cells overexpressing the Arylhydrocarbon receptor (AhR)

**DOI:** 10.1186/1471-2407-9-234

**Published:** 2009-07-15

**Authors:** Patrick S Wong, Wen Li, Christoph F Vogel, Fumio Matsumura

**Affiliations:** 1 Department of Environmental Toxicology and the Center for Environmental Health Sciences, University of California, One Shields Ave., Davis, CA 95616, USA

## Abstract

**Background:**

Recent reports indicate the existence of breast cancer cells expressing very high levels of the Arylhydrocarbon receptor (AhR), a ubiquitous intracellular receptor best known for mediating toxic action of dioxin and related pollutants. Positive correlation between the degree of AhR overexpression and states of increasing transformation of mammary epithelial cells appears to occur in the absence of any exogenous AhR ligands. These observations have raised many questions such as why and how AhR is overexpressed in breast cancer and its physiological roles in the progression to advanced carcinogenic transformation. To address those questions, we hypothesized that AhR overexpression occurs in cells experiencing deficiencies in normally required estrogen receptor (ER) signaling, and the basic role of AhR in such cases is to guide the affected cells to develop orchestrated cellular changes aimed at substituting the normal functions of ER. At the same time, the AhR serves as the mediator of the cell survival program in the absence of ER signaling.

**Methods:**

We subjected two lines of Michigan Cancer Foundation (MCF) mammary epithelial cells to 3 different types ER interacting agents for a number of passages and followed the changes in the expression of AhR mRNA. The resulting sublines were analyzed for phenotypical changes and unique molecular characteristics.

**Results:**

MCF10AT1 cells continuously exposed to 17-beta-estradiol (E2) developed sub-lines that show AhR overexpression with the characteristic phenotype of increased proliferation, and distinct resistance to apoptosis. When these chemically selected cell lines were treated with a specific AhR antagonist, 3-methoxy-4-nitroflavone (MNF), both of the above abnormal cellular characteristics disappeared, indicating the pivotal role of AhR in expressing those cellular phenotypes. The most prominent molecular characteristics of these AhR overexpressing MCF cells were found to be overexpression of ErbB2 and COX-2. Furthermore, we could demonstrate that suppression of AhR functions through anti-AhR siRNA or MNF causes the recovery of ERalpha functions.

**Conclusion:**

One of the main causes for AhR overexpression in these MCF breast cancer cells appears to be the loss of ERalpha functions. This phenomenon is likely to be based on the mutually antagonistic relationship between ER and AhR.

## Background

The arylhydrocarbon receptor (AhR), a ubiquitous basic Helix-Loop-Helix (bHLH) receptor expressed in various tissues in vertebrate species, is best known for its role in mediating the toxic actions of dioxin. On the molecular level it is known that: (a) dioxin binds to AhR, which exists in cytosol as a complex with a number of chaperone proteins, (b) the resulting dioxin-bound AhR migrates into nucleus where it forms a dimer with ARNT, another bHLH protein, (c) it is this dimer, which binds to the dioxin response element (DRE) on the promoters of the dioxin target genes, and (d) thereby causes induction of a number of detoxification enzymes. Tremendous efforts have been made in the past 25 years in elucidating the intricate molecular mechanisms through which this receptor accomplishes its tasks of inducing a number of detoxification enzymes and related proteins, particularly in the liver upon biding of dioxin and related environmental pollutants [1-3].

Recently, a surprising discovery has been made by David H. Sherr and his colleagues at Boston University that the AhR is overexpressed in DMBA-induced tumors *in vivo *in rats [4] as well as in mice [5]. Furthermore, the same group discovered that several *in vitro *cultured mouse as well as human breast cancer cell lines show high levels of expression of AhR [6]. In all these cases, the cells studied show the typical characteristics of advanced transformation. These scientists consider that in these cases, AhR itself must be playing the important tumor promoting roles in the development of mammary tumors even without the aid of its exogenous ligand [7]. Indeed artificial expression of AhRR, a specific "negative regulator" protein of AhR profoundly suppresses the growth of human mammary tumor cells as well as that of primary cultures of human thereby supporting the above notion that the presence of functionally-active AhR itself is the primary engine for these cells to maintain aggressive proliferation [8-10]. These landmark observations naturally have raised a new set of important toxicological questions; for instance, what the main cause for overexpression of AhR, how the overexpressed AhR contributes to those cellular changes even in the absence of its ligands, and how it contributes to malignant progression of those cells? These questions have prompted us to undertake the current investigation.

It is important to point out first that little is known about the natural physiological roles of AhR, which is defined as its active functions occurring in cells without the aid of exogenously introduced ligands, despite the colossal amounts of information available on the action of dioxins to activate AhR. Most of the clues for the possible physiological roles of AhR come from several studies on AhR-null (knockout) mice. It was originally reported from Chris Bradfield's group [11] that neonates of AhR-null mice develop liver abnormalities (e.g. in terms of reduced weight, and transient microvesicular fatty metamorphosis). Gonzalez *et al*. [12] have reported that their strain of AhR-null mice also show liver abnormalities which are accompanied with increased rates of apoptosis and increased production of TGF-β, probably due to excess retinoic acid accumulation. In the case of developing mammary epithelial tissues, it was reported by Hushka *et al *[13] that AhR-null mice show 50% reduction in the formation of terminal end buds (TEBs) in their mammary glands during estrous-stimulated growth and branding of ductal structures in the process of forming the lobule. Together, these observations implicate that there is a natural role of the AhR in coordinating animal development, cell growth, signaling of hormone receptors, and cytokine/growth factor production.

While the above observations on AhR-null mice have been helpful in demonstrating some of the consequences of AhR absence particularly during the development of animals, they by themselves do not indicate consequences of AhR overexpression in those mammary tumor cells. In this regard, one concrete example of the direct consequence of AhR overexpression has been provided by Andersson *et al*.[14], who produced a transgenic mouse strain that expresses a constitutively active AhR, and found that those mice developed distinct stomach tumors. This finding demonstrates that artificial overexpression of AhR is directly related to one type of tumor formation.

Based on the above observations, all implicating possible contribution of AhR on carcinogenesis, we have set our major objectives of this study as follows: first to identify at least a major cause for mammary epithelial cells to overexpress AhR, and second, to determine at least one essential role of AhR in those cells in expressing the phenotypic sign of advanced state of cell transformation.

## Methods

### Materials

Human recombinant EGF, serum and other media supplies were obtained from Gibco BRL (Gaithersburg, MD). [y-^32^P]ATP (4000 Ci/mmol) was purchased from ICN (Costa Mesa, CA). TCDD (>99.9% purity) was originally obtained from Dow Chemicals Co. 17-β-Estradiol (E_2_), and 4-OH-Tamoxifen (Tam) were obtained from Sigma-Aldrich Chemical Co. (St. Louis, MO). β-hexachlorocyclohexane (β-HCH) was purchased from Chem Service (Westchester, PA), Parthenolide, Resveratrol, and AG879 were obtained from Calibiochem (San Diego, CA). 3xERE-TATA-luciferase cDNA was a kind gift from D. McDonnell (Duke University Medical Center, Durham, NC. 3'-Methoxy-4'-nitroflavone (MNF) was a kind gift from Dr. Josef Abel (University of Düsseldorf, Institute of Environmental Research, Germany)

### Cell culture conditions for MCF10AT1 cell lines

MCF10AT1 cells were obtained from the Michigan Cancer Foundation, maintained in phenol red free Dulbecco's modified Eagle's medium with F12 nutrient mixtures (DMEM/F12; Gibco, BRL) and supplemented with 2.5% heat treated equine serum, 20 ng/mL EGF, 100 U/mL penicillin, and 100 μg/mL streptomycin at 37°C under 5% CO_2 _conditions. Cell culturing/selection was accomplished by seeding 1,000,000 cells (approximately 2.5% of a near confluent plate) into a 100 mm plate containing the above media and chemicals. In addition, selection chemicals (E_2_, 1 nM; Tam 10 nM; and β-HCH 1 μM final concentration) were added to the plates. Media and chemicals were refreshed twice a week and the plates were reseeded when plates reached 80–90 percent confluency (on average every 10–14 days). For the quantification of our long-term studies, each reseeding event constituted one passage.

### Proliferation test assay for MCF10AT1 cell lines

After 20 passages, cells were reseeded for cell proliferation assays. Cells were trypsinized from a starter plate and seeded at a concentration of approximately 50,000 cells per well in a 12 well plate. After 24 hours, the media was changed and one set of wells were trypsinized and counted with a hemocytometer to determine starting concentration of cells. It must be noted that none of the selection chemicals (i.e. E_2_, Tam or β-HCH were added to the cell culture media used during this assay). After an additional 48 hours, cells were trypsinized and counted with a hemocytometer. Trypan blue exclusion (0.08% dye) was used to determine viable cells (normally >96% of total cells).

### mRNA analysis of gene expression for MCF10AT1 cell lines

Cells that have been selected for 20 passages in our test chemicals were trypsinized and reseeded (500,000) into 60 mm dishes in our culture media. Note: no additional selection chemicals (i.e. E_2_, Tamoxifen, β-HCH) were added to the media during this analysis. After 24–48 hours, the media was refreshed with serum free media. For serum-free ("starved") media studies – following an additional 24 hours, test chemicals were added for an additional 3 hours and the mRNA was extracted from the cells. For "fresh" serum containing studies- following 21 hours from the last serum free media change, the cells were refreshed selection media described above, and chemicals were added 3 hours latter. The mRNA from these samples were then extracted 3 hours after chemical addition. For all cases mRNA extraction were completed using the Qiagen (Valencia, CA) RNAeasy kit. cDNA was prepared from the extracted mRNA using the Omniscript Reverse Transcriptase Kit (Qiagen) and analyzed by real-time PCR on a Roche lightcycler.

### Electrophoretic Mobility Shift Assays (EMSA)

Nuclear extracts were isolated from MCF10AT1 cells as described by to Dennler *et al *[15]. In brief, MCF10AT1 cells were washed and harvested in Dulbecco's PBS containing 1 mM phenylmethylsulfonyl fluoride (PMSF) and 0.05 μg/μl aprotinin. After centrifugation the cell pellets were gently resuspended in 1 ml of hypotonic buffer (20 mM HEPES, 20 mM NaF, 1 mM Na_3_VO_4_, 1 mM Na_4_P_2_O_7_, 1 mM EDTA, 1 mM EGTA, 0.5 mM PMSF, 0.13 μM okadaic acid, 1 mM dithiolthreitol, pH 7.9, and 1 μg/ml each leupeptin, aprotinin, and pepstatin). The cells were allowed to swell on ice for 15 min and then homogenized by 25 strokes of a Dounce homogenizer. After centrifugation for 1 min at 16,000 *g*, nuclear pellets were resuspended in 300 μl of ice-cold high salt buffer (hypotonic buffer with 420 mM NaCl and 20% glycerol). The samples were passed through a 21-gauge needle and stirred for 30 min at 4°C. The nuclear lysates were microcentrifuged at 16,000 *g *for 20 min, aliquoted, and stored at -70°C. Protein concentrations were determined by the method of Bradford [16]. For EMSA, a double-stranded oligonucleotide corresponding to the DRE binding site or end-labeled using [y-^32^P]ATP (Amersham Life Sciences) and T4 polynucleotide kinase (Promega, Madison, WI) according to the standard methods. DNA-protein-binding reactions were carried out in a total volume of 20 μl containing 15 μg of nuclear protein, 40,000 cpm of DNA oligonucleotide, 25 mM Tris buffer, pH 7.5, 50 mM NaCl, 1 mM MgCl_2_, 1 mM EDTA, 0.5 mM dithiolthreitol, 5% glycerol, and 1 μg of poly (dI-dC). Supershift analysis was performed by adding 2 μg of monoclonal ARNT, polyclonal RelB (Active Motif), or polyclonal AhR (Novus Biologicals) antibodies to the reaction mixtures. Competition experiments were performed in the presence of a 100-fold molar excess of unlabeled DNA fragments. The samples were incubated at room temperature for 20 min. Protein-DNA complexes were resolved on a 5% non-denaturating polyacrylamide gel and visualized by exposure of the dehydrated gels to x-ray films. In all cases each EMSA test was repeated more than two times to ascertain the reproducibility of the overall pattern of nuclear protein binding to the labeled oligonucleotides.

### Antibodies and Western blotting

A polyclonal anti-human AhR (SC-5579), a polyclonal antibody against human ACTIN (SC-1616), a horseradish peroxidase conjugated secondary antibody, and pre-stained standard markers (SC-2361) were obtained from Santa Cruz Biotechnology (Santa Cruz, CA). Whole cell lysates (30 μg) were separated on a 10% SDS-polyacrylamide gel and blotted onto a PVDF membrane (Immuno-Blot, Bio-Rad, Hercules, CA). The antigen-antibody complexes were visualized using the chemiluminescence substrate SuperSignal^®^, West Pico (Pierce, Rockford, IL) as recommended by the manufacturer. For quantitative analysis, respective bands were quantified using a ChemiImager™ 4400 (Alpha Innotech Corporation, San Leandro, CA).

### ERE-Luciferase experiments

For transient transfection experiments, cells were plated in 6-well culture plates (5 × 10^4^/well). After 24 hrs, cells were washed once with PBS, and 1.6 ml of fresh serum/phenol red free growth medium was added before transfection complexes were applied drop by drop to the cells. Cells were transiently transfected for 16 hrs by using 10 μl per well Effectene (Qiagen, Valencia, CA) with 0.5 μg per well of respective luciferase reporter constructs of the 3 xERE-TATA promoter according to the manufacturer's instructions. The serum and phenol-red free media was then refreshed. After an additional 24 h incubation period, the cells were washed twice with PBS and lysed with 300 μl of passive lysis buffer. Luciferase activities were measured with the Luciferase Reporter Assay System (Promega, Madison, MI) using a luminometer (Berthold Lumat LB 9501/16, Pittsburgh, PA). Relative light units were normalized to protein concentration, using a Bradford dye assay (Bio-Rad). Experiments were repeated three times and three wells of cells were analyzed per experiment.

### Apoptosis Detection by Annexin V Staining

Cells were seeded at 1 × 10^5 ^cells in 60 mm culture dishes in 2 ml medium after 24 hrs media was refreshed. After 21 hrs, cells were treated with various inhibitors and incubated for and additional 3 hrs. Apoptosis was induced by UV light (100 μJ/cm^2^) for 100 seconds and incubated at 37°C for 4 hrs. All media except for 200 μl was removed from each plate. Afterwards, 2 μl CaCl_2_(20 mM), 2 μl annexin V-fluorescein isothiocyanate (50 μg/ml, Sigma) and 2 μl of propidium iodide (PI) solution (50 μg/ml) were added directly to each plate. The cells were gently mixed and incubated for 10 minutes at room temperature in the dark. Apoptotic cells were counted directly by using the fluorescence microscope (Leitz, Wetzlar, Germany). In each experiment, ten representative fields were counted for apoptosis assay. Both annexin V-positive and annexin V-PI-double-positive cells were considered to be apoptotic.

### Statistical Analysis

All quantitative experiments were repeated a minimum of three times and results are expressed as means ± standard deviations. Data were evaluated statistically by one-way ANOVA followed by Student's *t *test at the significant level of *P *< 0.05.

## Results

### Genesis of AhR over-expressing MCF cells and their characterization

**Figure 1:**
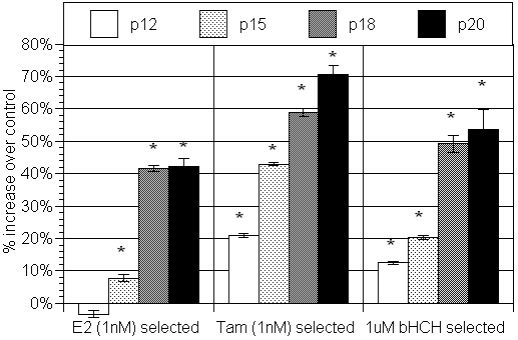
**Cell proliferation of MCF10AT1 cells selected over 12–20 passages in the presence of 17-β-estradiol (E_2_), 17-β-Tamoxifen (Tam) or β-hexachlorohexane (β-HCH)**.

**Figure 2:**
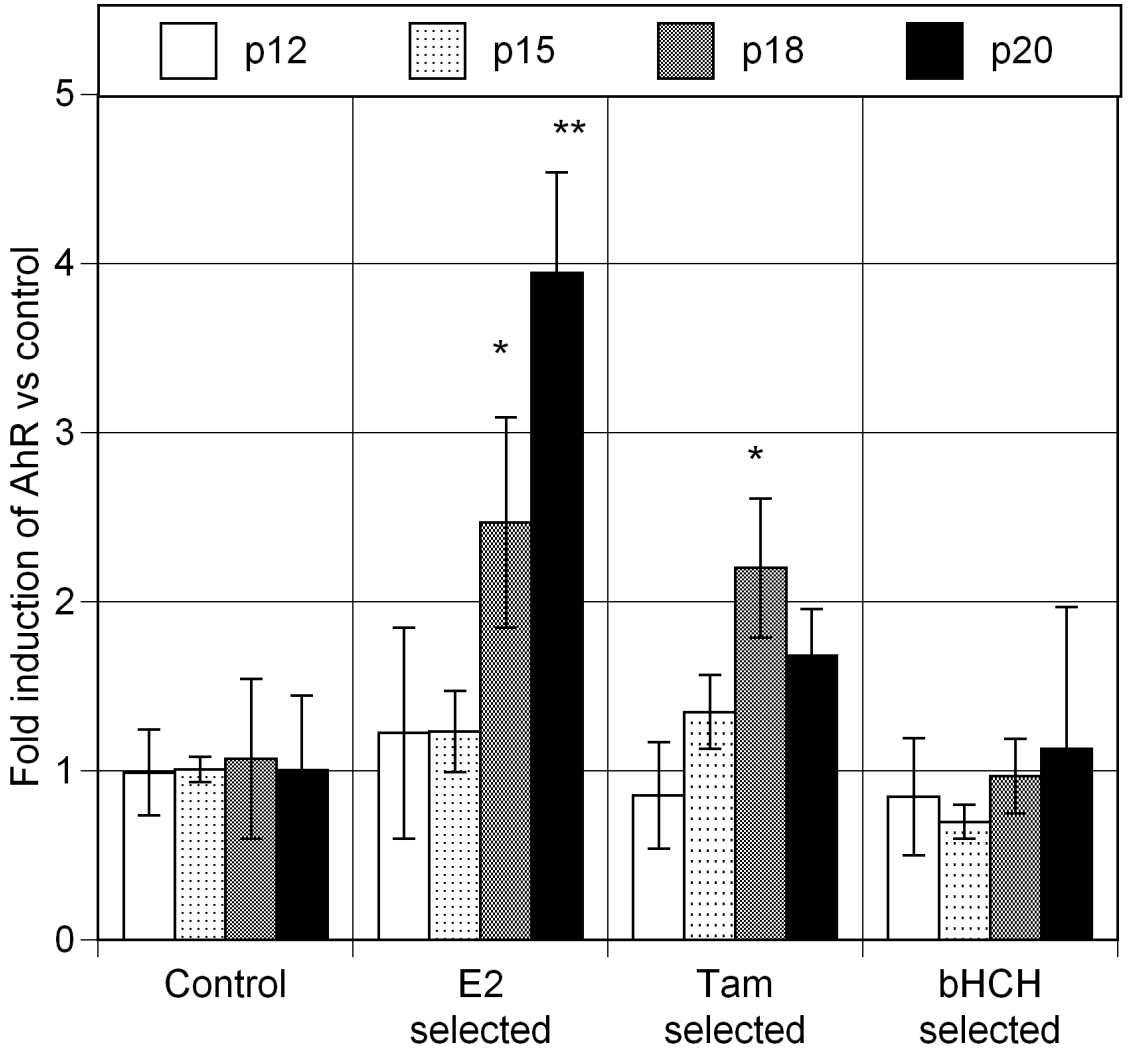
**AhR expression in MCF10AT1 cells selected for various passages in the presence of 17-β-estradiol (E_2_), 4-OH-Tamoxifen (Tam) or β-hexachlorohexane (bHCH)**.

**Figure 3:**
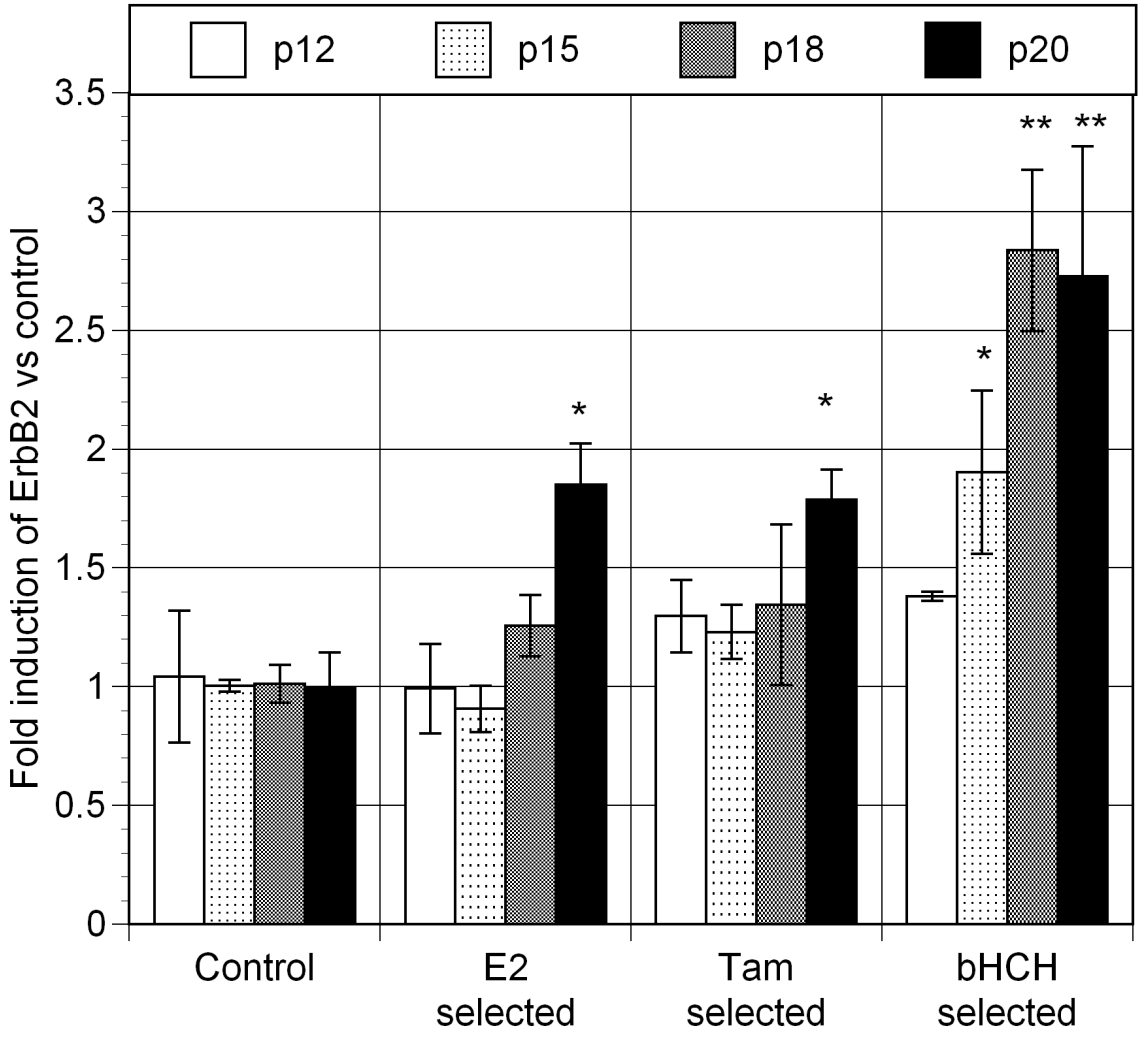
**ErbB2 expression in MCF10AT1 cells selected for various passages in the presence of 17-β-estradiol (E_2_), 4-OH-Tamoxifen (Tam) or β-hexachlorohexane (bHCH)**.

To address the question on the possible cause(s) of AhR overexpression in mammary epithelial cells, we began this project by exposing MCF10AT1 cells to three estrogen receptor interacting chemicals, 1 nM E_2_, 10 nM 4-hydroxy-tamoxifen, or 1 μM β-hexachlorohexane (β-HCH), an estrogenic pesticide, throughout a number of passages under our standard culture conditions using "selection medium" which was phenol red-free and contained heat-inactivated serum (2.5%) [17]. After 20 passages, those selected cells have exhibited distinct signs of increased proliferation, when tested in "selection medium" (Figure 1). To gain insight into the main cause for such phenotypic changes in each of selected cell line, we have tested the effectiveness of two well known chemical inhibitors, AG879, a specific inhibitor for ErbB_2 _associated tyrosine kinase, and 3-methoxy-4-nitroflavone (MNF), a specific antagonist to the Ah receptor (AhR) on those cells (Table 1). It was found that MNF was particularly effective in suppressing the rate of proliferation of E_2 _selected lines, while AG879 was more effective in this regard on those selected by 4-hydroxy-tamoxifen (Tam) or by β-HCH (Table 1), suggesting perhaps that AhR plays an important role in inducing increased proliferation of the E_2_-selected line, and that contribution of ErbB_2 _may be more important in Tam- and β-HCH- selected cells than E_2_-selected ones in terms of promoting cell proliferation. This possibility was further checked by examining the changes in mRNA expressions of AhR (Figure 2) and ErbB_2 _(Figure 3) in these 3 sub-lines during their selection process. It is apparent from this study that the expression of AhR is indeed outstanding in E_2_-selected cells (Figure 2), and that the elevated expression of ErbB_2 _mRNA is most pronounced in β-HCH selected cells followed by those selected by Tam (Figure 3). In general, those selected MCF10AT1 cells passages showed the highest expression of AhR and ErbB_2 _mRNAs after 20 passages, except in the case of AhR expression in Tam-selected cells, and ErbB_2 _expression in β-HCH selected ones, both of which showed the sign of decline of their expression in 20 passage from that was found in 18 passage. In view of this observation, we have decided not to subject those cells for further selection by any of these selecting agents beyond 20 passages in the case of MCF10AT1.

**Table 1:** Effects of inhibitors on the cell proliferation of MCF10AT1 sub-lines selected in the presence of 17-β-estradiol (E_2_), Tam and β-Hexachlorohexane (β-HCH) *p < 0.05 vs. control ^a^p < 0.05 vs. no chemicals.

	Control	E_2_	Tam	β-HCH
Time 0	100 ± 6.8	100 ± 5.0	100 ± 12.1	100 ± 11.9

After 48 hours:				
No chemicals	787 ± 65	1274 ± 61*	1456 ± 56.9*	1378 ± 138*
AG879	263 ± 16	1025 ± 51*	283 ± 13.^a^	535 ± 12* ^a^
MNF	810 ± 60	850 ± 4.3^a^	1355 ± 52.2*	1425 ± 125*

### Comparison of selected MCF10AT1 cells to similarly selected MCF-7 cells

**Figure 4:**
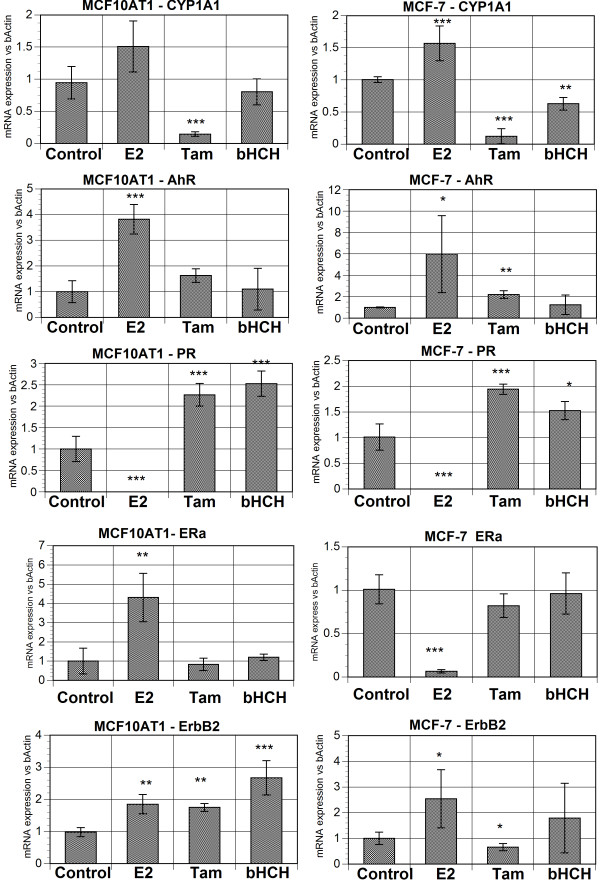
**mRNA expression of selected proteins in long-term selected cells – MCF10AT1 and MCF-7 cell lines grown in the presence of 17-β-estradiol (E2, 1 nM), 4-OH-Tamoxifen (Tam, 10 nM) and β hexachlorohexane (β-HCH, 1 μM) for 20 and 34 passages respectively**. PR = progesterone receptor. * p < 0.05 ** p < 0.01 ***p < 0.001 vs control

Since one of the main objectives of this study was to investigate the cause for AhR overexpression in breast cancer cells, we wanted to have at least another example of mammary epithelial cells developing AhR overexpression for the purpose of confirming the effect of E_2_. Fortunately we have already conducted a similar experiments on MCF-7 cells which had been selected by the same set of agents utilizing essentially identical procedures for 35 generations [18]. At that time, however, we had no knowledge on the expression of AhR or its consequences in those selected MCF-7 cell lines. The results of qRT-PCR assessments of selected marker mRNA expressions in these two E_2_-selected lines of MCF10AT1 and MCF-7 (Figure 4) show that for the given treatments, the basic mRNA expression profile of these two different cell lines are remarkably similar. For instance, in both cell lines, E_2_-selection induced the highest level of mRNA expression of AhR followed by those selected by 4-OH-tamoxifen (Tam) and β-HCH. A parallel assay on CYP1A1 expression, which is being used here as a marker for the functional activation of AhR, indicated that, while E_2_-selected ones (i.e. P_35_E and P_20_E for MCF-7 and MCF10AT_1_) still showed the highest expression among all sub-lines within each MCF cell group, β-HCH selected cells (as compared to "mock" selected control cells) exhibit also relatively higher expressions of the ratio of CYP1A1/AhR than Tam-selected ones. This set of data indicated that the level of induction of AhR is not exactly identical to that of CYP1A1, implying that the functional activation of AhR may be governed by a different set of cellular conditions or factors than its mRNA induction. Nevertheless, in both cases, E_2_-selected cell lines showed clearly the highest levels of AhR and CYP1A1 mRNA expressions among all selected lines. The observation that E_2_-selection in both MCF10AT1 and MCF-7 cell lines resulted in AhR overexpression and an increased proliferation indicates that estrogen signaling suppression might play a dominant role in these processes. In addition, these observations indicate some growth factor signaling such as that mediated by ErbB_2 _or their downstream signaling cascades are also likely to play important roles as well. As for the status of the estrogen receptor (ER), these two E_2_-selected cell lines show some different characteristics (Figure 4). E_2_-selected MCF-7 line (P_35_E) was found to show a drastically low mRNA level of ERα, but E_2_-selected MCF10AT_1 _line (i.e. P_20_E) did not show such a decrease in the mRNA expression of ERα as compared to the matched "mock"-selected control cell line (designated as P_20_C) (not shown). However, P_20_E MCF10AT1 cells showed no detectable level of expression of either progesterone receptor (PR) as in the case of P_35_E (shown in Figure 4) or PS2 (not shown). Such an observation suggests that in both cell lines ER functions are likely suppressed. The difference between these two cell lines appears to be that, in the case of P_20_E it is ER signaling that is impaired, but in the case of P_35_E it is the ERα mRNA titer that is suppressed.

In the case of ErbB_2 _mRNA, both E_2_-selected and 1 μM β-HCH-selected cells in both cell lines showed high expression ErbB_2 _(= c-Neu or HER2). Of great interest to this study was the differential upregulation of AhR and ErbB_2 _in cell lines selected by E_2_, 4-OH-tamoxifen and β-HCH respectively. The differences among them might represent different routes through which MCF10AT1 cells could be affected by these chemicals to develop transformation. An interim conclusion we have reached from this follow-up study are: (a) E_2 _selection resulted in the greatest up-regulation of AhR mRNA expression, (b) β-HCH, on the other hand, greatly increases mostly ErbB_2 _mRNA expression, and (c) 4-OH-tamoxifen produces an intermediate type of cells showing modest increases in the expression of both of these markers. These findings support our notion that AhR overexpression is a likely result of alteration of the status of the estrogen receptor as a result of exposure to excess E_2 _or to 4-OH-tamoxifen.

### Effects of chemical and siRNA suppressors of the function of AhR, and ErbB_2 _on proliferation of P_20_E cells in comparison to P_20_C cells

To investigate the possibility that AhR may be a contributing factor for increased cell proliferation in this transformed mammary epithelial cell type, we re-examined the influence of several diagnostic inhibitory agents on P_20_E and P_20_C cells. The results summarized in Table 2 show that MNF, an AhR antagonist, is indeed a powerful agent suppressing the rate of cell proliferation in P_20_E cells both in the presence and the absence of exogenous heregulin (HRG). In addition, the anti-inflammatory agent parthenolide was also quite effective in suppressing proliferation. Resveratrol, which has both AhR antagonist [19,20] and anti-inflammatory properties [21], was the most effective in proliferation suppression particularly in P_20_E cells. To confirm the role of AhR on cell proliferation further, we have tested the effectiveness of two siRNA preparations (Table 3), i.e. siAhR and si-β-catenin and compared them to neg-siRNA (Qiagen -"Allstar"). The si-β-catenin was used as a positive control to suppress cell proliferation that are mediated by this wnt/β-catenine pathway, which might or might not include the one mediated by AhR. The results showed that, while both siRNA preparations were effective in suppressing proliferation of both cell lines, siAhR appears to be more effective in equalizing the rate of proliferation between these two lines of cells in culture.

**Table 2:** Proliferation in MCF10AT_1 _cell sublines selected over 20 passages in 17-β-Estradiol (P_20_E_2_) or the ethanol vehicle (P_20_C).

Cell lines:	P_20_C	P_20_E
Control	100 ± 10	224 ± 11
+ Resveratrol (20 μM)	91 ± 15	123 ± 12*;
+ Parthenolide (10 μM)	98 ± 15	183 ± 8.5*
+ MNF (10 nM)	103 ± 6.3	178 ± 8.8*
+ HRG (1 ng/ml)	108 ± 8.0	295 ± 12^a^
+ HRG + MNF	108 ± 7.4	184 ± 9.2

Cells were measured 72 hrs after plating. * p < 0.05 inhibitors vs. control, ^a ^p < 0.05 HRG vs. control, ^b ^<0.05 HRG+MNF vs. HRG

**Table 3:** Effect of AhR and β-Catennin siRNA on proliferation in MCF10AT1 cell sub-lines selected over 20 passages in 17-β-Estradiol (p20 E2) or the ethanol vehicle (p20 C).

Cell lines:	P_20_C	P_20_E
Control neg-siRNA	100 ± 6.3	195 ± 10.1* ^a^
β-Catenin siRNA	60 ± 4.2^a^	103 ± 12.5* ^a^
AhR siRNA	63 ± 4.2^a^	86 ± 8.7* ^a^

Cells were measured 72 hours after plating. * p < 0.05 HRG vs. control, ^(a)^p < 0.05 control vs. siRNA

### Studies on the contribution of the over expressed AhR on resistance to induced apoptosis in P_20_E cells compared to P_20_C cells

**Figure 5:**
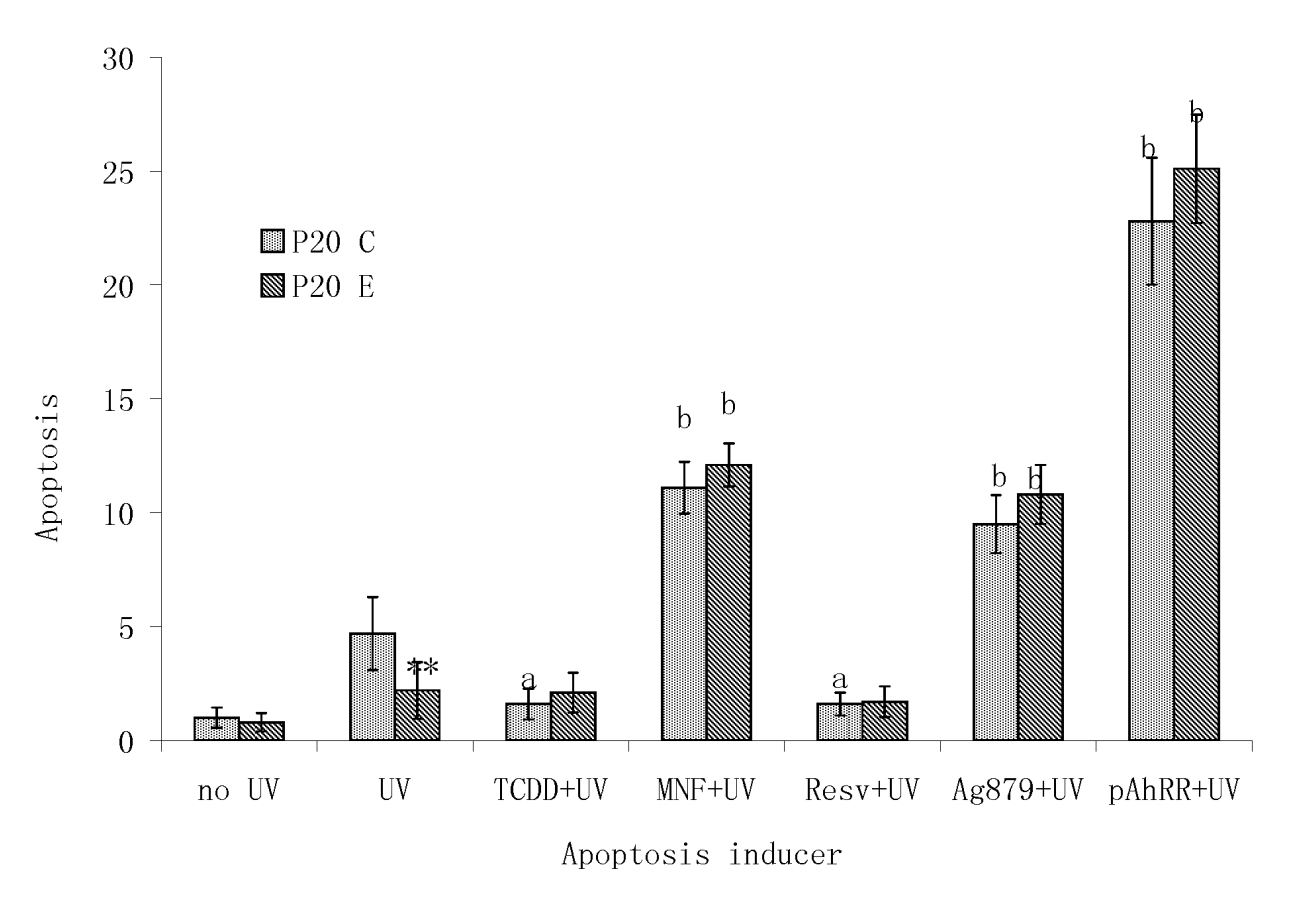
**Differential effects of apopototic action of UV-irradiation between P_20_C and P_20_E cells, and the influence of AhR affecting, diagnostic agents in synergizing or antagonizing the effect of UV-irradiation**. ** the difference between P_20_C and P_20_E was significant at p < 0.01. ^b ^the effect of MNF, AG879, Resveratrol (Resv) or pAhRR, as compared to UV alone, was significant at p < 0.001.

**Figure 6:**
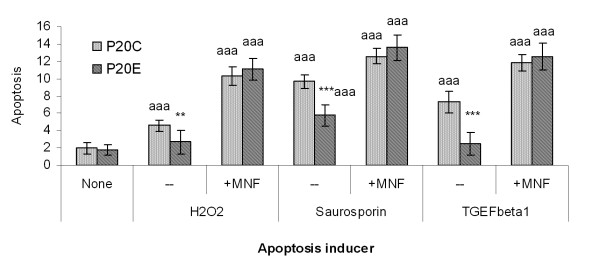
**Differential effects of apoptosis-inducing action of hydrogen peroxide (H2O2), staurosporin and TGFβ1 and their dependency on the function of AhR**. *,**,*** the difference between P20C and P20E was significant at p < 0.05, 0.01 or 0.001, respectively. ^a, aa, aaa^The effect of each treatment, as compared to "None", was significant at p < 0.05, 0.01 or 0.001, respectively.

To assess the contribution of AhR in conferring P_20_E cells another typical phenotype of transformation, we have selected apoptosis resistance. In the first series of experiments apoptotic stimuli were provided by externally applied ultraviolet (UV)-irradiation on cells maintained under a modestly serum-deprived culture conditions (i.e. 24 hrs serum deprivation) (Figure 5). This UV-treatment induced approximately 5-fold increase in apoptosis under our test conditions in P_20_C, but only 2.5-fold increase in P_20_E. TCDD treatment induced an anti-apoptotic reaction as shown previously [22], but mostly on P_20_C only. All suppressors of AhR consistently induced marked increases of apoptosis. Resveratrol, which is known to have both agonistic and antagonistic action on AhR, on the other hand, acted like TCDD in this case suppressing apoptosis in both cell lines. To test the possibility that this contribution of AhR confers apoptosis resistance to more than one type of apoptosis inducers, or not, we tested the effects of H_2_O_2_, staurosporin and TGFβ1 on both cell lines either in the presence or the absence of MNF (to suppress the function of AhR) (Figure 6). Each of apoptosis inducer caused the increase in the number of cells showing apoptosis as expected, but their actions were invariably less pronounced in P_20_E cells than in P_20_C cells. Again the effect of MNF was to further increase the number cells showing apoptosis, and in its presence the difference between P_20_C and P_20_E cells disappeared.

### Tests on the AhR-dependency of expression of selected mRNA markers

At this stage we have decided to investigate the contributions of the overexpressed AhR on the expression of several diagnostic markers under both fresh serum (3 hrs after the medium change with fresh serum) and serum starved (72 hrs after the last medium change with serum). For this purpose we conducted additional qRT-PCR studies on the expression of selected mRNA markers in P_20_E and P_20_C cells both in the presence and the absence of MNF to gain insight to the differential contribution of AhR in expressing each of these marker expressions between these two cell lines. The results (Table 4) showed that unexpectedly CYP1A1 expression was much higher in both cell lines under serum starved conditions than fresh serum conditions. On the other hand, as expected its expression was higher in P_20_C cells than in AhR overexpressing P_20_E, which was more apparent under serum starved conditions. MNF, a blocker of AhR was effective in significantly suppressing the expression of CYP1A1, indicating the functional activation of AhR even in the absence of TCDD or any other exogenous ligands. The expression of AhR mRNA itself, on the other hand, was not drastically affected by the culture conditions of those cells or by MNF. In comparison, both ErbB_2 _and TGF-β1 showed a higher levels of expression under serum starved conditions than in fresh serum conditions as in the case of CYP1A1, except that their sensibility to MNF was apparent only in P_20_E cells. In contrast TGF-α and heregulin (HRG) expression was higher under fresh serum conditions than in serum starved conditions. On the other hand, the effects of serum or MNF on PI3K were not significant. This set of experiments established that the contributions of AhR to the expression of those selected markers are greatly affected by the condition of serum in culture medium. It appears at the same time, there are at least 2 different types of types of markers: (a) those expression is higher under fresh serum conditions (e.g. TGF-α and HRG), and (b) those favor serum starved conditions (CYP1A1 and TGFβ1). The AhR dependency of those mRNA expressions, as judged by the blocking action of MNF, is mostly confined to P_20_E compared to P_20_C (except in the case of CYP1A1 and AhR under serum starved conditions), and is more noticeable under the condition where the given mRNA expression is high (e.g. HRG and TGFα under fresh serum, and TGF-β3, ErbB_2 _and, to a lesser extent, ErbB2 under serum starved conditions) than the conditions promoting low expression.

**Table 4:** Effect of serum starvation of P_20_C and P_20_E cells on the expression of selected marker mRNAs.

	Fresh serum				Serum starvation			
	
mRNA	P_20_C		P_20_E		P_20_C		P_20_E	
	
	C	MNF	C	MNF	C	MNF	C	MNF
**CYP1A1**	1.00	1.25	1.21	1.03	28.38**^bb^	10.18**^abb^	41.16**^bb^	25.36**^abb^

**AhR**	1.00	1.03	2.11*	1.74*	2.02**^bb^	1.46**^bb^	1.43**	1.67*

**ErbB2**	1.00	1.68*^a^	4.24**	2.07*^a^	4.24**^bb^	5.81**^bb^	5.41***^bb^	3.21*^ab^

**HRG**	1.00	1.84*^a^	11.82**	3.47*^a^	0.82	0.93^b^	0.51**^bb^	0.24**^ab^

**PI3K**	1.00	1.00	2.67*	2.64**	1.51**^bb^	1.90*^b^	3.72**^bb^	4.27***^bb^

**TGF-α**	1.00	1.10	1.49	1.18	0.98	0.80^bb^	0.23**^bb^	0.28**^bb^

**TGF-β3**	1.00	2.04**^a^	4.96***	2.87*^a^	7.03*^b^	7.65**^bb^	12.61**^bb^	8.41**^ab^

Statistical symbols are: * different from control (C = 1.00) fresh serum P_20_C, ^a ^significant effect of MNF from the matched control, ^b ^significant effect of serum starvation. One symbol indicates P ≤ 0.05; two P ≤ 0.01; three P ≤ 0.001.

Serum/medium was given 3 hrs prior to the test designated as "fresh medium." For "serum starvation" tests, the same batch of cells treated with fresh serum/medium were given serum-free medium after 48 hrs and the samples were harvested 24 hrs thereafter.

### Studies on the influence of the overexpressed AhR on the mRNA expression of selected inflammation markers in P_20_E cells as compared to P_20_C cells

One of the characteristic functions of AhR is to mediate the action of dioxin to induce cellular inflammatory responses [23]. Preliminary test results showed that the expressions of inflammation markers are higher under fresh serum conditions than starved ones. Accordingly in this sub-project we have tested the possibility of the overexpressed AhR affecting the inflammatory status of P_20_E cells under fresh serum conditions only. The results summarized in Table 5 indicate that markers that are differentially affected by MNF in these two cell lines are COX-2, CSF-1, and MCP-1. In addition, the markers affected by MNF only in P_20_E cells were NOX-2 (NADPH oxidase 2), and NFkB. Two anti-inflammatory agents, NS389 (a COX-2 inhibitor) and quercetin (a bioflavonoid with overall anti-inflammatory/anti-oxidant activities) were used to assess the extent of their participation in causing the cellular state of inflammation. The result showed that, as expected, these two agents are reasonably effective in suppressing the expression of NFκB. In addition, they generally reduced the expression of other markers to roughly the similar extents as those achieved by MNF. These findings support the rationale of selecting these mRNAs as the markers for inflammation. They also indicated that the expression of these markers are definitely higher in P_20_E than in P_20_C, and that, judging by the suppressive effect of MNF, AhR contributes to their increase expression to certain extent.

**Table 5:** mRNA expression of selected inflammatory and oxidatives markers under "Fresh serum" conditions (see methods).

	Fresh Serum					
	
mRNA	P_20_C			P_20_E		
	
	C	MNF	C	MNF	NS398	Quercetin
NFκB	1.00	1.30	2.25***	2.00	1.52^a^	1.45^aa^

MCP-1	1.00	0.80	4.07***	3.07	2.21^aa^	1.01^aaa^

CSF-1	1.00	0.84	3.77***	2.66***^a^	3.45	5.92^a^

COX-2	1.00	0.72	5.67***	2.72***^a^	3.60^aa^	2.11^aaa^

NOX-2	1.00	1.10	3.75***	2.49***^a^	3.97	2.85^a^

### Electromobility Shift Assay (EMSA) study on AhR binding to the dioxin response element (DRE) in P_20_E in comparisons to P_20_C cells

**Figure 7:**
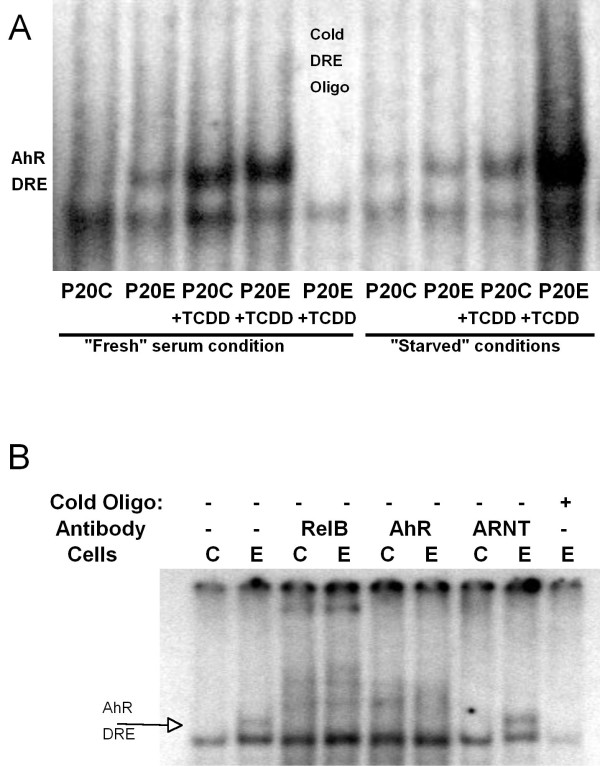
**EMSA on Dioxin response element (DRE) Activation**. Nuclear proteins from P_20_C and P_20_E cells grown under "fresh" serum and serum starved conditions alone or in the presence of TCDD (10 nM-3 hrs) were hybridized with a DRE-containing ^32-^P labeled oligonucleotide.

To ascertain the functional importance of the overexpressed AhR on the increased expression of CYP1A1(previously illustrated in Figure 4 top left), which is known to be activated by AhR:ARNT dimmer binding to dioxin response element (=DRE), an EMSA analysis was conducted on nuclear proteins isolated from samples of P_20_C and P_20_E cells under fresh serum (6 hrs after medium change with fresh serum) and serum starved (72 hrs after the last serum change/24 hours since last media change) conditions. In addition, as a positive control, the effect of TCDD (10 nM) was also determined under these conditions to confirm the presence of the AhR:ARNT-DRE complex. The results in Figure 7A have revealed that nuclear protein binding to the DRE sequence of oligonucleotides is clearly higher in the samples obtained from the overexpressed AhR in P_20_E cells than those prepared from "mock-selected" P_20_C cells. This observation indicates that these DRE binding proteins (likely including AhR) are functionally active in terms of their DRE binding even without the presence of the exogenously added ligand under this test condition. Thus, in this system, overexpressed AhR confers P_20_E cells the additional influence of functionally active AhR over that is found in P_20_C. Interestingly the effect of TCDD appears to be more pronounce in cells under serum starved conditions, while the extent of protein binding to the DRE sequence in the absence of TCDD is actually higher in serum fresh conditions than starved conditions. This finding suggests that under fresh serum conditions the level of constitutively activated AhR function in P_20_E, even in the absence of exogenously added ligands, is already very high (i.e. higher than that is found in P_20_C exposed to 10 nM of TCDD). To test the specificity of AhR functionality, gel-shift (i.e. supershift) assays were performed onP_20_C and P_20_E cells under fresh serum conditions (Figure 7B). Recent studies in our laboratory have demonstrated that the NFκB subunit RelB can crosstalk and dimerize with AhR to activated DRE [24-26] especially in the absence of an AhR ligand. Therefore it was not surprising that both the AhR and RelB antibody resulted in the loss of AhR/DRE complex (Figure 7B) and appearance of new supershift bands. The ARNT antibody which does not affect the AhR/DRE complex under these conditions acts as a negative-antibody control.

### Western blotting studies on the protein expression of AhR in P_20_E and P_20_C cells

**Figure 8:**
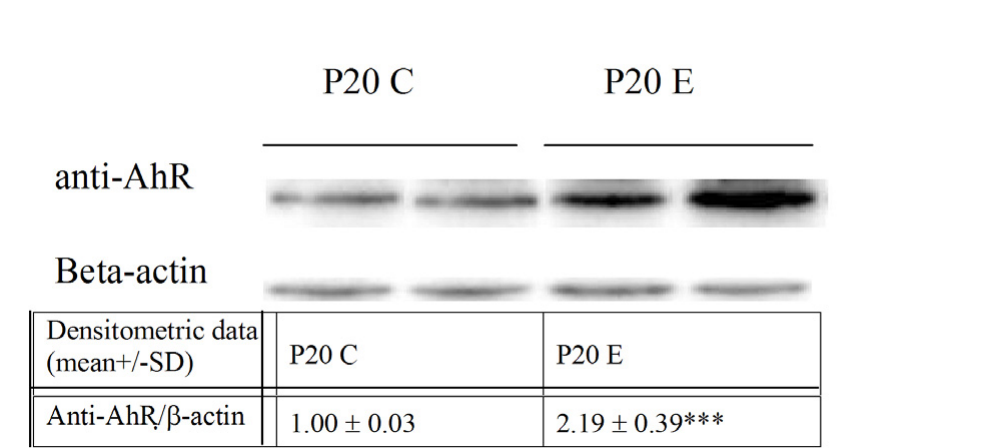
**Western blot for AhR protein expression in P_20_C vs P_20_E cells**.

Since overexpression of AhR in P_20_E cells in comparison to P_20_C cells has only be studied so far by its mRNA expression and by assessing the parallel increase in CYP1A1 mRNA expression, we conducted a western blot test to assess the titer of its protein in these two strains of cells. Indeed, we could confirm the higher AhR protein expression in P_20_E cells than that in P_20_C cells (Figure 8).

### Studies on the effect of AhR on the expression of ERE-target genes through the use of ERE-Luc reporter plasmid

**Figure 9:**
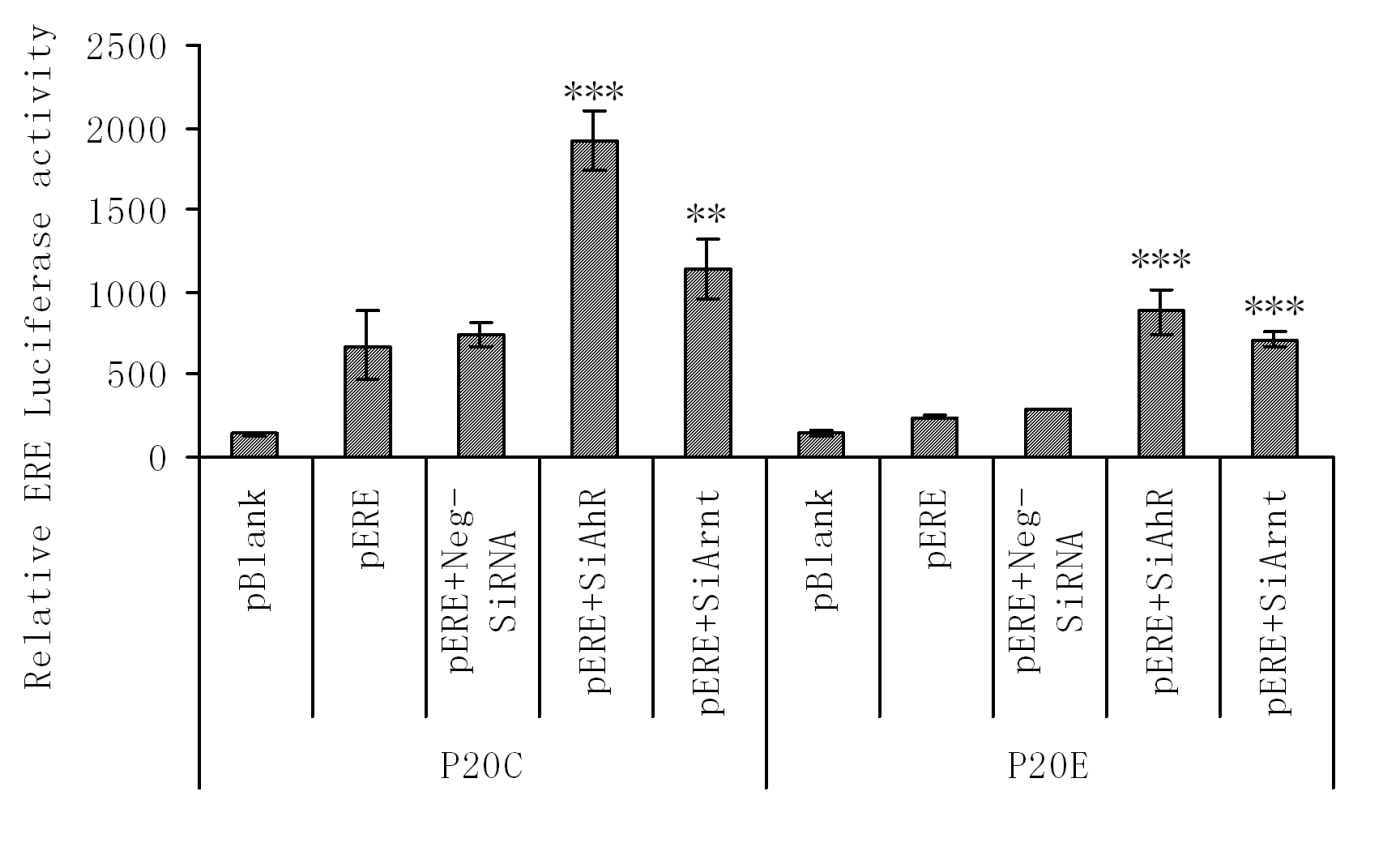
**Differential level of expression of Estrogen Response Element (ERE)-dependent gene activation between P_20_C and P_20_E cells, and assessment of the effectiveness of siAhR and siArnt to interfere the function of AhR and Arnt, respectively as determined via pERE-Luc reporter assays on P_20_C vs. P_20_E cells**. **,*** The effect of siAhR or siArnt treatment, as compared to "scrambled RNA" treated sample in each strain of cells, were significant at p < 0.01 or 0.001, respectively.

In view of the lack of the difference in the level of expression of ERα protein between these two cell lines, we hypothesized that the actual difference may be in the function of ERα itself. To test this hypothesis, we assessed the levels of estrogen response element (ERE)-mediated gene expression in P_20_E and P_20_C cell lines using an ERE-Luciferase (ERE-luc) reporter plasmid transfection approach. The result (Figure 9) clearly showed that the level of constitutive expression of the luciferase activity is significantly lower in P_20_E cells than that in P_20_C. Furthermore, treatment of these cells with an siRNA against AhR (siAhR) or that against ARNT (a required dimerization partner for the AhR target gene activation) clearly caused up-regulation of the luciferase expression, indicating that AhR and its dimer with ARNT work antagonistic to the nuclear transcription factors (likely including the ER) binding to ERE as positive regulators.

## Discussion

Our main strategy for the current study has been first to identify the most likely cause for these MCF breast cancer cells to overexpress AhR by checking the effectiveness of three estrogenic agents in stimulating the expression of AhR for a number of passages, and second to identify phenotypic signs of transformation along the process of selecting those cells by these agents. This approach has yielded two major tangible results: (a) in our main study cell lines, MCF10AT1 (i.e. P_20_E) as well as in MCF-7(P_35_E) cells, E_2 _was the most effective agent, among tested in terms of eliciting AhR overexpression, and (b) E_2 _selection resulted in transformed cells with a phenotypic expression of the increased cell proliferation in both cell lines. The above finding suggests that the estrogen receptor (ER) is likely involved in this process of AhR up-regulation at least in these cell lines under our test conditions. In the case of MCF-7 cells we found the mRNA expression of ERα itself in E_2_-selected cells (P_35_E) is totally suppressed. On the other hand in the case of E_2_-selected MCF10AT1 cells (P_20_E) the extent of down-regulation of expression of ERα mRNA, in comparison to P_20_C cells, was not relatively modest. Instead we found that the level of mRNA expressions of progesterone receptor (PR) and PS2, both estrogen receptor element (ERE) controlled proteins, were very low, indicating possibly that the function of the ERα is impaired in P_20_E as a result of its overexpression of AhR. In this regard, the results of ERE-Luciferase (ERE-Luc) assays (Figure 9) were most helpful in confirming the above diagnosis, since both siRNA treatments aimed at reducing the expression of AhR caused the restoration of ERE-Luc activity in both cell lines. This indicates that there is definitely a good possibility of the existence of an antagonistic influence of AhR on the expression of genes promoted by ERE. Furthermore, even in the absence of siRNA, the level of ERE-dependent gene expression in P_20_C was found to be significantly higher than that in P_20_E. One unexplainable question remaining is why the extent of the effect of those siRNA treatments in recovering ERE-dependent gene expression is more pronounced in P_20_C than in P_20_E: i.e. if the degree of AhR dependency of ERE-suppression is higher in P_20_E than P_20_C, the expected outcome of suppression of AhR would be more in the former. One possibility, which we consider likely to explain this observation, is that this phenomenon is due likely to the incompleteness of this siRNA treatment (estimated to be effective in suppressing 70% of AhR mRNA expression). In other words, even the remaining AhR still expressed in P_20_E cells is powerful enough to keep the expression of the ERE-target genes suppressed. An alternate possibility is that there is another factor other than AhR which makes the functional activation of ERE less effective in P_20_E cells than in P_20_C. The third possibility, which is even less likely, is that the transfection efficiency of these cells with the ERE-Luc plasmid was compromised due to molecular differences between the two sublines. Nevertheless, the main conclusion derived from these experiments that the functions of ERα and ERE are reduced in P_20_E cells in comparison to P_20_C is still firm, clearly supporting our conclusion.

The second major objective has been to find the effect of the overexpressed AhR on the increased expression of at least one phenotype indicating the AhR-dependent progression to more advanced state of transformation. It must be noted that MCF-7 P_35_E line, in comparison to its matched control P_35_C line, has already been shown to exhibit several phenotypic characteristics of further advanced state of transformation such as accelerated anchorage-independent growth in soft agar, increased invasiveness, as judged by matrigel invasion tests, and cell proliferation [17]. These changes were accompanied with marked suppression of expressions of ERα and significant increase in that of MMP-9 both at protein and enzymatic levels [17]. Since much less is known about MCF10AT1 derived P_20_E cells than those selected from MCF-7 (i.e. P_35_E versus P_35_C cells), we decided to study MCF10AT1 as our main cellular model in examining this phenomenon of AhR overexpression in more detail. In this regard, the most noticeable finding of the current study has been that AhR overexpression through E_2_-selection is accompanied with increased cell proliferation in both MCF cells lines. This observation merits special attention, since increased cell proliferation is clearly one of the major phenotypic expressions of advanced transformation of mammary epithelial cells. The most significant pieces of evidence procured for the critical contribution of AhR in helping P_20_E cells to express the above transformation characteristics were first, the effectiveness of MNF, a specific blocker of AhR and second, that of siRNA against AhR in effectively suppressing cell proliferation of P_20_E cells. Thus, by this approach we could satisfactorily meet the second objective of this project to unequivocally show, at least in one case, that AhR indeed contributes significantly to the progression of transformation of mammary epithelial cells. To support the above diagnosis of the influence of AhR on progression of cellular transformation, we have further conducted the apoptosis resistance study, which clearly showed that AhR overexpression is intimately associated with the phenotypic expression of apoptosis resistance in P_20_E cells.

The third topic needing discussion is the relationship between AhR overexpression in these cells and their inflammatory status. The extent of contribution AhR on the expression of these inflammation markers was assessed by the effectiveness of MNF in reducing the mRNA expression of those markers (Table 4). However, the extent of suppression achieved by MNF was not complete in all cases. Judging by the effectiveness of parthenolide, an anti-inflammatory agent, in reducing the proliferation difference between P_20_E and P_20_C, on the other hand (Table 2), there is likely possibility that inflammation itself is contributing to the expression of this phenotype. Nevertheless, at this early stage of investigation, the contribution of the overexpressed AhR on the increased expression of the inflammation status in P_20_E cells is still based on this circumstantial evidence only, and therefore this subject still remains to be resolved in the future.

One final topic needing a brief discussion is how the elevated AhR in MCF cells becomes functionally activated even without addition of exogenous ligands. It must be pointed out that we avoided the use of typical ligands such as TCDD throughout this study (except in Figure 7A). Yet, the result of this current study indicates unambiguously that overexpressed AhR in P_20_E cells are functionally active even without exogenous ligands (e.g. see Figure 7 and 9). Therefore, while we could not address this specific question in this study, this topic deserves a serious attention in the future.

## Conclusion

We could clearly establish in the current study that E_2_-selection causes induction of AhR overexpression in two MCF breast cancer cell lines, and that such a phenomenon is accompanied with increased cell proliferation that is significantly dependent on the presence of AhR. The evidence suggests that the basic cause for up-regulation of AhR by E_2_-selection is related to the E_2_-induced down-regulation of either expression of ERα as in the case of MCF-7 cells, or the loss of the function of estrogen responsiveness in MCF10AT1 cells.

## Abbreviations

AhR: Arylhydrocarbon Receptor; ARNT: Arylhydrocarbon nuclear transferase; DRE: dioxin response element; EMSA: Electrophoretic Mobility Shift Assay; ER: estrogen receptor; E_2_: 17-β-estradiol; ERE: Estrogen Response Element; HRG: heregulin; MCF: Michigan Cancer Foundation; MNF: 3-methoxy-4-nitroflavone; qRT-PCR: quantitative-Real-Time – Polymerize Chain Reaction; TCDD: 2,3,7,8-Tetrachlorodibenzo-p-Dioxin.

## Competing interests

The authors declare that they have no competing interests.

## Authors' contributions

This study was conceived jointly by both PSW and FM. PSW was responsible for experimental design and completion of all laboratory work unless noted contained in this article. EMSA data (Figure 7) was completed in conjunction with CV and LW. LW was responsible for the laboratory work and data preparation regarding apoptosis and reporter gene assays (Figures. 5, 6, 8, &9). FM, PSW, CV participated in the design and coordination of the work involved. The manuscript was drafted by PSW. All authors approve and read the final manuscript

## Pre-publication history

The pre-publication history for this paper can be accessed here:

http://www.biomedcentral.com/1471-2407/9/234/prepub
